# A comprehensive guide to conduct a systematic review and meta-analysis in medical research

**DOI:** 10.1097/MD.0000000000041868

**Published:** 2025-08-15

**Authors:** Ernesto Calderon Martinez, Patricia E. Ghattas Hasbun, Vanessa P. Salolin Vargas, Oxiris Y. García-González, Mariela D. Fermin Madera, Diego E. Rueda Capistrán, Thomas Campos Carmona, Camila Sanchez Cruz, Camila Teran Hooper

**Affiliations:** a Facultad de Medicina, Universidad Nacional Autónoma de México, Mexico City, Mexico; b Facultad de Medicina, Universidad Católica de Honduras, Tegucigalpa, Honduras; c Facultad de Medicina, Universidad Westhill, Ciudad DE México, México; d Facultad de Medicina, Instituto Tecnológico y de Estudios Superiores de Monterrey, Jalisco, México; e Facultad de Medicina, Instituto Tecnológico de Santo Domingo, Santo Domingo, República Dominica; f Facultad de Medicina, Universidad de Ciencias Médicas, San José, Costa Rica; g Facultad de Medicina, Universidad Mayor de San Simón, Cochabamba, Bolivia.

**Keywords:** meta-analysis, research, systematic review

## Abstract

Systematic reviews and meta-analyses are essential tools in medical research. Systematic reviews are a type of literature review that uses a systematic process to identify and assess all available literature on a specific research question. A meta-analysis is a statistical method of synthesizing the results of a systematic review by quantitatively combining data. The process begins with formulating a well-defined research question using frameworks. Comprehensive literature searches across multiple databases, including PubMed, Embase, and Cochrane, to ensure the inclusion of diverse studies. Tools like EndNote and Covidence streamline reference management and study selection, enhancing efficiency and accuracy. Quality assessment using tools like the Cochrane Risk of Bias Tool and Newcastle-Ottawa Scale is crucial to evaluate the methodological rigor. Data extraction, using standardized forms to ensure consistent information capture. Qualitative synthesis is one method that integrates the results of a systematic review focusing on textual data. Meta-analysis employs statistical software such as R and RevMan to compute effect sizes, confidence intervals, and assess heterogeneity. Visual representations, including forest and funnel plots, facilitate the interpretation of results. Challenges such as publication bias and heterogeneity are addressed using statistical methods like Egger regression and the trim-and-fill technique. Sensitivity analyses further validate the robustness of findings. Common errors, including data entry mistakes and inappropriate pooling, are mitigated through rigorous methodological adherence and critical self-evaluation. Meticulously conducted, systematic reviews and meta-analyses represent the pinnacle of the evidence hierarchy, driving advancements in medical research and practice.

## 1. Introduction

There is an increased need for medical professionals and researchers to stay up to date with the literature due to the constant expansion of current evidence.^[1]^ Integrating high-quality research findings is vital to evidence-based medicine, and doing so frequently means going through a large number of primary studies that need to be carefully reviewed and synthesized in order to produce correct interpretations. In response to this difficulty, systematic reviews have become an essential component of secondary research.^[2]^ They use scientific techniques to compile, evaluate, and summarize all pertinent research on a certain subject. By using a systematic approach, the bias present in individual studies is reduced, making systematic reviews a more reliable source of information. The primary goal is to support transparent, objective, and repeatable healthcare decision-making while guaranteeing the validity and reliability of the results.^[3]^ Systematic reviews can address research questions that require the review of qualitative or mixed-methods studies, which combine both quantitative and qualitative data. In such reviews, qualitative data is extracted and synthesized from the included studies. A qualitative study is one that uses qualitative methods of data collection and analysis (interviews, focus groups, case studies, surveys, etc) to provide a narrative view of the topic being studied.^[4]^ A systematic review serves as the basis for a meta-analysis, in which the results of the selected studies are combined, and statistical pooling is performed.^[5]^ Meta-analysis enhances the accuracy of estimates and offers an overall view of the impacts of interventions. This increases the study’s power and, per se, the viability of the results.^[6]^ When a meta-analysis is not possible or appropriate, researchers can use other synthesis methods, such as qualitative synthesis, a method that combines data from multiple qualitative studies, or synthesis without meta-analysis (SWiM).^[7]^ Historically, meta-analysis has its roots in early statistical work from the 1930s and gained popularity in the 1970s when it was used to synthesize results from several studies in various topics, including psychology and medicine.^[8]^ The process became especially useful in situations when the results of individual investigations were contradictory or unclear. Systematic reviews and meta-analyses have gained popularity because they offer empirically supported responses to specific topics of study, giving crucial information to guide future clinical research and patient care, therefore, it is essential to be capable of performing them and understanding how to interpret them. This article’s primary goal is to provide the reader with the fundamental steps that support systematic reviews and meta-analyses.

## 2. Formulating the research question

Every systematic review or meta-analysis needs to establish a well-defined research question to ensure a structured approach and analysis. Establishing the inclusion and exclusion criteria will permit a more efficient process. Therefore, frameworks are different tools that are designed to formulate an organized research question in qualitative mixed-methods and quantitive research.^[8]^ They can be divided according to 10 types of reviews, each focused on specific research question and different framework. These include: e*ffectiveness reviews* (evaluates the treatment outcome), *experiential reviews* (explores personal experiences), *cost/economic evaluation reviews* (assessing cost-effectiveness), *prevalence/incidence reviews* (measures prevalence/incidence rates), *diagnostic accuracy reviews* (evaluates test performance), *etiology/risk reviews* (evaluates particular exposures/risks and outcomes), *expert opinion/policy reviews* (synthezing experts opinion), *psychometric reviews* (evaluates measurement of psychometric properties of certain tests), *prognostic reviews* (assessing factors influencing outcomes), and *methodical reviews* (analyzing research methods). Each of the review types follows a specific question format (e.g., PICO, PICo, CoCoPop) adapted to its focus.^[9]^

Among these instruments, the most frequently used frameworks are PICO (Population, Intervention, Comparator, Outcome) or its extension, PICOTTS (Population, Intervention, Comparator, Outcome, Time, Type of Study, and Setting) (see Table 1),^[10–13]^ and SPIDER (Sample, Phenomenon of Interest, Design, Evaluation, and Research Type), which are particularly for therapy-related question and can also be adapted for diagnosis and prognosis. Other less-used formats are SPICE (Setting, Perspective, Intervention/Exposure/Interest, Comparison, and Evaluation) and ECLIPSE (Expectation, Client, Location, Impact, Professionals, and Service).^[8,10,14]^The SPICE framework is useful in project proposals and quality improvement where the outcome or intervention is evaluated because it assesses the setting where it occurs, whose perspective is being considered, and how the change (intervention) made works. On the other hand, the ECLIPSE tool is valuable for research questions evaluating the outcomes of healthcare policies and services by including all key components like the goal, people involved, setting, and who is delivering the service.^[10]^ The PICO framework is mainly focused on therapy questions. Still, due to its adaptability, research questions can also be centered on diagnosis and prognosis, making it the most popular among investigators.^[10,11,15]^

**Table 1:** Structure of PICOTS framework.^[10–13]^

	Structure	Meaning	Example 1^[12]^	Example 2^[13]^
P	Population/patient/ problem	People that is planned to be affected (age group, socio-demographic characteristics, duration of disease, and severity).	Adults > 18 yr old with portal hypertension	General population
I	Intervention	Medicines, procedures, health education, public health measures, preventive measures like vaccination and prophylaxis. It can also be called “Exposure” abbreviated as (E). Exposure can be depicted by diagnostic tests, prognostic markers, and prevalence of conditions.	Not applicable	Silymarin supplements
C	Comparison	Gold standard treatment, placebo or an alternative intervention. Some study designs or research questions don’t demand to include comparison.	Not applicable	Placebo/standard treatment
O	Outcome	The result that intervention (I) has on the population (P) compared to comparison (C). Most systematic reviews focus on efficacy, safety and sometimes cost. When focused on diagnostic tests, the goal is to measure accuracy, reliability and cost. It can be dichotomic or continuous.	Predictive factors for Bleeding of esophageal varices	Improvement in liver enzymes (ALT, AST, ALP)
T	Time-frame	Outcomes become relevant only when assessed in a specific period of time.	Not applicable	Not applicable
T	Type of study	Specific protocol that allows the conduction of the study. Have risk of bias which increases in the following order: RCT, nonrandomized trials, cohort studies, case-control studies, case series, and case reports.	Observational, cohort, case-control studies	Randomized control trials
S	Setting	The location where study takes place. It can be primary, specialty, inpatient, outpatient, or nursing home among others.	Not applicable	Not applicable

Abbreviation: RCT = randomized controlled trial.

Regularly, systematic reviews and meta-analyses will specify the research question in the introduction section or will provide enough information to the reader to structure the framework. In case this information is not given, there is an increased risk of biased interpretation. Additionally, if the Population (P) is too detailed, the study has the risk of limited generalizability.^[10]^ A well-defined research question should provide clear guidance on each stage of the process by helping identify relevant studies and establishing inclusion and exclusion criteria in the literature search, determining the relevant data information in the data extraction, and guiding the integration of data from different studies in the data synthesis.^[16]^

## 3. Literature search strategy

### 3.1. Sources to search

Once we have created our research question it is time to start working into the literature search for systematic reviews and/or meta-analyses, this should be gathered in bibliographic databases, which provide access to vast scientific articles.^[17]^ Multiple online databases can be used in the literature search, such as Embase, MEDLINE, Web of Science, and Google Scholar, among others. The choice of the databases to use should be based on the research topic with the aim to obtain the largest amount possible of studies relevant to the research question. At least 2 of these databases should be used in the search.^[2]^ Moreover, including published and unpublished studies (known as gray literature) will reduce the risk of publication bias, resulting in more exact diagnostic accuracy in meta-analysis and higher chances for exploring the causes of heterogeneity.^[2,10]^ Reference managers, such as Zotero and Mendeley, or Endnote, can be used to collect the searched literature, remove duplicates, and manage the initial list of publications. Tools like Rayyan and Covidence can also help in the screening process. Covidence assists in the study screening, data extraction and quality improvement; on the other hand, Rayyan aids in the screening section by suggesting inclusion and exclusion criteria and allowing collaboration from other members.^[2]^ Additionally, understanding the fundamental characteristics and purpose of the various tools and databases will help the researcher conduct a more comprehensive and efficient research (see Table 2).^[14,18–27]^

**Table 2:** Main characteristics of databases.

Database	Main characteristics
PubMed^[14,18]^	Free platform that allows the use of MEDLINE, which is a database that provides life sciences and biomedical database. It is maintained by the National Library of Medicine and allows the use of Boolean operators and MeSH terms.
EMBASE^[14]^	Biomedical and pharmacological database by Elsevier B.V. that covers drug, pharmacology, toxicology, dependence and abuse, clinical and experimental medicine, health care, forensic medicine and biotechnology topics.
Cochrane^[19]^	Database of systematic reviews and meta-analyses.
Google Scholar^[19]^	Free access engine that allows scholar literature search of articles, theses, books, abstracts, court opinions from academic publishers, professional societies and universities among others.
Web of Science^[20]^	Platform for citation reference search. Provides access to Science Citation Index Expanded (SCI-EXPANDED), social sciences citation index (SSCI), and arts & humanities citation index (A&HCI).
Science Direct^[21]^	Database that offers books and journals in areas of physical science, engineering, life, health, social sciences and humanities.
PsychINFO^[22]^	Electronic database that provides abstracts and citations on psychology, social, behavioral, and health science.
ICTRP^[23]^	Portal that offers access to information about ongoing and completed clinical trials.
Clinical Trials^[24]^	Database provided by the U.S National Library of Medicine of privately and publicly funded clinical studies from around the world.
LILACS^[25]^	Online platform coordinated by Pan-American Health Organization that provides scientific index and technical literature of Latin-American and Caribbean with access to community and health professionals.
CNKI^[26]^	China National Knowledge Infrastructure Database. Multidisciplinary database of more than 3500 journals published in China.
SCOPUS^[27]^	Database produced by Elsevier that provides abstracts and citations with full-text links.

### 3.2. Keywords and search terms

**Figure 1.**
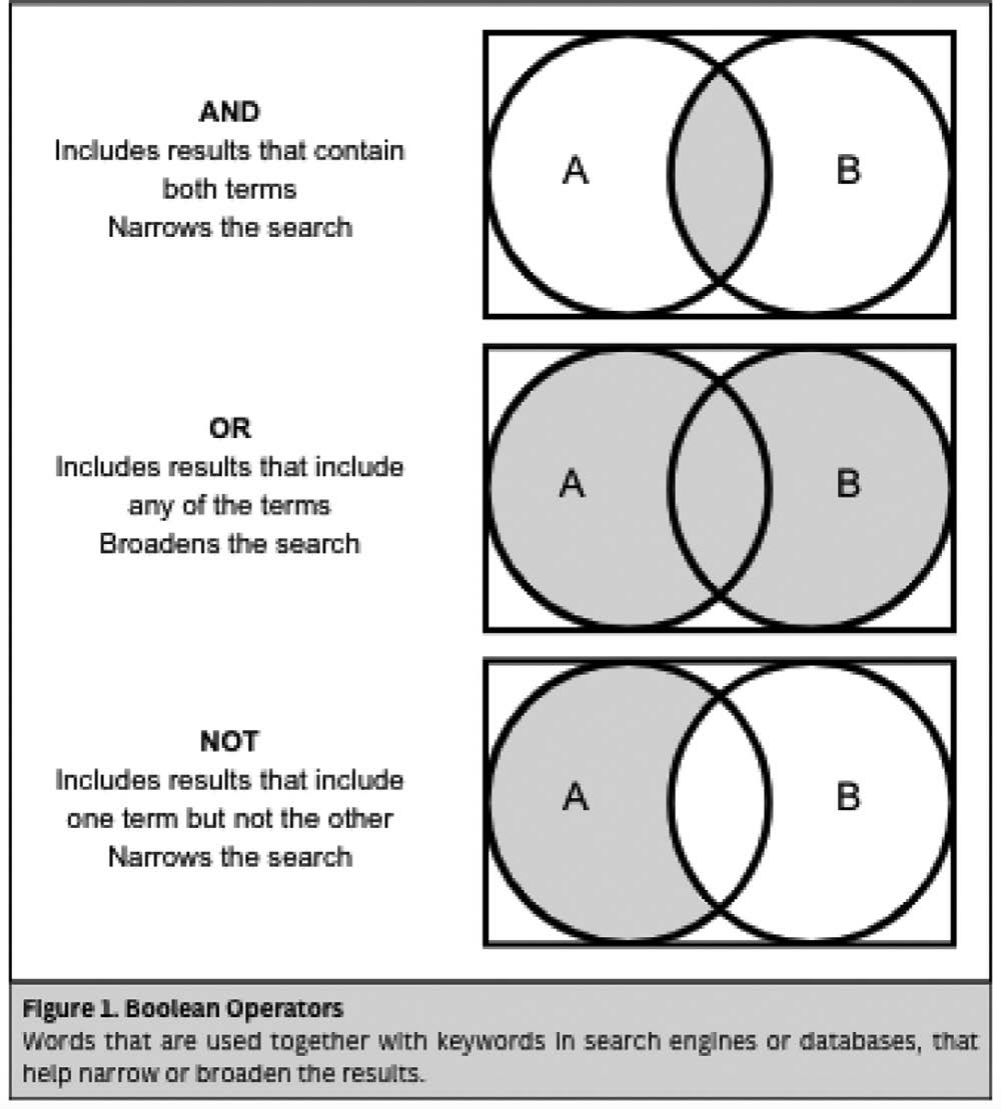
Boolean operators: words that are used together with keywords in search engines or databases, that help narrow or broaden the results.

For a systematized search strategy, different methods for search, like assorted terms and Boolean operators, could be used (see Fig. 1). First, the investigator needs to identify key concepts and terms related to the research question. Then, for each key term, identify synonyms, related terms, variations, abbreviations, misspellings, singulars, and plurals.^[14]^ MeSH terms (Medical Student Headings) are a controlled and systematized vocabulary produced by the National Library of Medicine (NLM) with the objective of categorizing health-related information. They include subject headings that PUBMED can use to search in MEDLINE.^[28]^ Each MeSH term includes synonyms, for example, “Aspirin” and “Acetylsalicylic Acid”.^[29]^ It is important to notice that we need to develop a singular search for each database we include as some won’t accept Boolean operators or MeSH terms, but despite this the search need to be equally balanced among all the databases.^[14]^

## 4. Methodology

Systematic reviews and meta-analyses rely on stringent study selection criteria to ensure the reliability and impact of synthesized research findings. Defining clear inclusion and exclusion criteria is crucial to pinpoint studies directly relevant to the research objectives, enhancing transparency and validity. Collaboration with experienced librarians is essential from the outset to develop robust search strategies across key databases like CENTRAL, MEDLINE, and Embase. Eligibility criteria, guided by the research question, ensures rigor and objectivity in study selection. Criteria are documented in the review protocol to facilitate transparency and adjustments based on emerging data.^[30]^

Validated search filters, such as those for randomized trials, are utilized to optimize search efficiency. However, filters often perform differently across databases; a filter effective in PubMed may produce less results in Embase due to differences in indexing and terminology. Using filters inappropriate for the research question, such as an RCT filter for diagnostic accuracy studies, can result in the omission of crucial literature. Filters may also introduce biases, such as emphasizing English-language studies or excluding older but relevant research, which could limit the diversity of evidence. To use filters effectively, they should align with the study design and context, be tested for performance against known reference studies, and be combined with manual screening to minimize missed results. Filters are best applied when focusing on specific study designs or when precision is essential.^[17]^

## 5. Standardized data extraction forms

Data extraction, also referred to as data collection or abstraction, involves systematically organizing and extracting information from each included study. This process ensures consistency and thoroughness in capturing pertinent data across studies. The use of standardized data extraction forms or templates is pivotal in maintaining uniformity in data collection.^[30]^ During data extraction, it is imperative to identify and document key data elements relevant to the systematic–review or meta-analysis. These typically include study characteristics (e.g., author, year, country), participant demographics (e.g., age, gender), intervention specifics (e.g., type, dosage), outcomes evaluated (e.g., primary and secondary outcomes), and reported results (e.g., effect sizes, *P*-values**).**^[31]^ Effective data management is essential for maintaining accuracy and accessibility of extracted data throughout the systematic review process. Utilizing appropriate tools such as Excel, JBI Sumari, Covidence, or systematic review management software facilitates secure data handling.^[32,33]^ Transparent documentation of data extraction methods ensures reproducibility and supports future updates or data sharing initiatives.^[34]^

## 6. Protocol development

### 6.1. Definition of protocol development and importance

Protocol development is critical for ensuring methodological rigor and transparency in research. A well-crafted protocol serves as a structured roadmap, foundational for enhancing the reliability and reproducibility of study findings.^[35]^ It minimizes bias by guiding systematic evidence synthesis and decision-making processes during study selection and data extraction, thus optimizing resource utilization and credibility by providing a structured framework that ensures methodological consistency.^[30,36]^ Transparency is enhanced through a clear delineation of objectives, methods, and analytical approaches, which are crucial for deriving evidence-based conclusions.^[37]^

### 6.2. Key protocol sections

The preferred reporting items for systematic reviews and meta-analyses (PRISMA) guidelines provide a set of evidence-based items to report in systematic reviews and meta-analyses, aiming to enhance transparency and consistency in the reporting process. PRISMA-P, a version specifically for protocols, emphasizes detailed administrative information, a clear rationale and study objectives, specific criteria for study selection, and the search strategy. On the other hand, the Cochrane guidelines, developed by the Cochrane Collaboration, are widely recognized as a gold standard for conducting systematic reviews. They focus on a structured approach that includes sections such as title, background, detailed methods for study selection and data analysis, and consumer involvement, ensuring rigor and comprehensiveness in the review process. Key protocol sections therefore vary significantly between PRISMA-P and Cochrane guidelines, reflecting their distinct emphases and methodological approaches (See Table S1, Supplemental Digital Content, https://links.lww.com/MD/O720, which illustrates the difference between PRISMA-P and Cochrane).^[30,37]^

### 6.3. Protocol registration and integrity

Protocol registration, such as in International Prospective Register of Systematic Reviews (PROSPERO), is crucial for methodological transparency and reducing bias. PROSPERO is an open-access database where researchers can register their systematic review protocols, which helps in documenting study plans, promoting accountability, and preventing deviations from the initial methodology. Early registration in PROSPERO fosters collaboration, enhances resource efficiency, and supports the rigorous conduct of systematic reviews.^[38]^

### 6.4. Choosing a registration platform

There are several platforms available for protocol registration, but researchers often select specific ones based on their focus. For example, PROSPERO is commonly used for health reviews, Research Registry is preferred for health and medicine, and OSF Registries cater to interdisciplinary studies. Platforms like Figshare and VTechWorks offer general accessibility and visibility (See Table S2, Supplemental Digital Content, https://links.lww.com/MD/O720, which describes and explains the advantages of these platforms for registering systematic review protocols).^[39–43]^

### 6.5. Benefits of registration

Prospective registration enhances systematic review credibility for clinical decisions and policy. Adherence ensures research reliability, method transparency, and stakeholder collaboration, upholding integrity and evidence robustness in reputable databases like PROSPERO.

### 6.6. Adherence to protocol in reviews

Strict adherence to protocol criteria ensures methodological rigor and credibility. Transparent method reporting supports reproducibility and comparability, promoting evidence synthesis quality. Despite challenges, maintaining protocol integrity advances research excellence.

## 7. Study selection screening process

### 7.1. Screening process

Once we have registered our protocol we need to run our search strategy and extract all the information form the databases to start with the first step in initiating the systematic review and meta-analysis process, which involves deduplicating references by identifying and removing duplicate records to streamline the review process and ensure each study is evaluated only once, as previously mentioned tools such as references managers (EndNote, Mendeley, or Zotero) and specific tools for Systematic reviews (Rayyan or Covidence) can help to eliminate de-duplication.

The next stage is title and abstract screening, where each study’s title and abstract are reviewed by 2 independent reviewers to determine if they meet predefined eligibility criteria.^[2]^ Disagreements between the 2 reviewers are resolved by a third reviewer to increase scientific rigor and ensure a balanced assessment.^[14]^ After the title and abstract screening, the full text of all references that passed this initial screening is located and obtained for a detailed evaluation against the inclusion criteria specified in the systematic review protocol. The full-text review is also conducted by 2 independent reviewers, with disagreements resolved by a third reviewer, and reasons for excluding studies that do not meet the criteria are documented. If a full-text PDF is unavailable, it is recommended to use public libraries or inter-library loan services. Contacting the original author should only be considered if it complies with copyright laws. Additionally, researchers can search for further references in previous studies or cited studies to locate the necessary documents. While tools like Rayyan, Covidence, and DistillerSR are primarily designed for systematic review management, they can support the organization and tracking of studies identified through citation searching.^[32,44,45]^ Each of these stages, from initial duplication to detailed full-text review, is crucial to ensure that the systematic review process is rigorous, transparent, and based on objective criteria. This contributes to the quality and reliability of the synthesized findings.

### 7.2. Tools for screening

Covidence is recommended for managing references, customizing screening processes, and generating PRISMA diagrams. Alternative tools like Rayyan, Abstrackr and ASReview offer additional screening solutions (see Table 3)^[32,33,44,46,47]^

**Table 3:** Comparing screening tools.

Tool	Pros	Cons	Costs	Special features
COVIDENCE^[32]^	Efficient automation for title and abstract screening. Supports collaborative screening and data extraction.	High cost relative to other tools. Limited to 5 reviewers on the basic plan.	$289 USD/yr (basic plan). Additional fees for more users or features.	Provides bulk upload of references, integrates with citation managers like EndNote, and offers conflict resolution tools for reviewers.
Rayyan^[44]^	Highly sensitive and specific for title screening in systematic reviews. User-friendly interface.	Free version is limited to small projects; full functionality requires a paid subscription. Some features, like advanced collaboration tools and full integration with reference managers, are only available in the paid version.	Free for basic use; pricing starts at $100/yr for premium features.	Utilizes AI-driven text mining methods for semiautomated screening. Allows for blinding and unblinding of reviewer decisions, and offers tagging and coding options.
Abstrackr^[46]^	Facilitates semiautomated abstract screening with a user-friendly interface. Provides objective scoring in reviewer assessment.	Limited functions: lacks full-text screening, citation management integration, and advanced reporting features.	Free	Uses predictive modeling to suggest the relevance of abstracts. Can be used for collaborative reviews, though it may require more manual effort for conflict resolution.
ASReview^[47]^	Uses active learning to prioritize records for review, improving efficiency in identifying relevant studies.	Limited features beyond screening, such as no built-in citation management or advanced data extraction tools.	Free, Open-source software.	Incorporates machine learning algorithms for iterative screening, which adapts based on reviewer input to improve the relevance of subsequent records.
JBI SUMARI^[33]^	Offers an intuitive and aesthetically pleasing dashboard, guiding users through each stage of the systematic review process with large clickable buttons and fillable boxes.	The software does not support title and abstract screening, also does not allow for the upload of full-text studies, focusing instead on final reviews.	$130 USD per annum. First-time users can access a 14-d free trial to evaluate the software’s features	JBI SUMARI supports 3 different types of data synthesis, simplifying the creation of forest plots and meta-aggregative flowcharts.

### 7.3. PRISMA flow diagram

**Figure 2.**
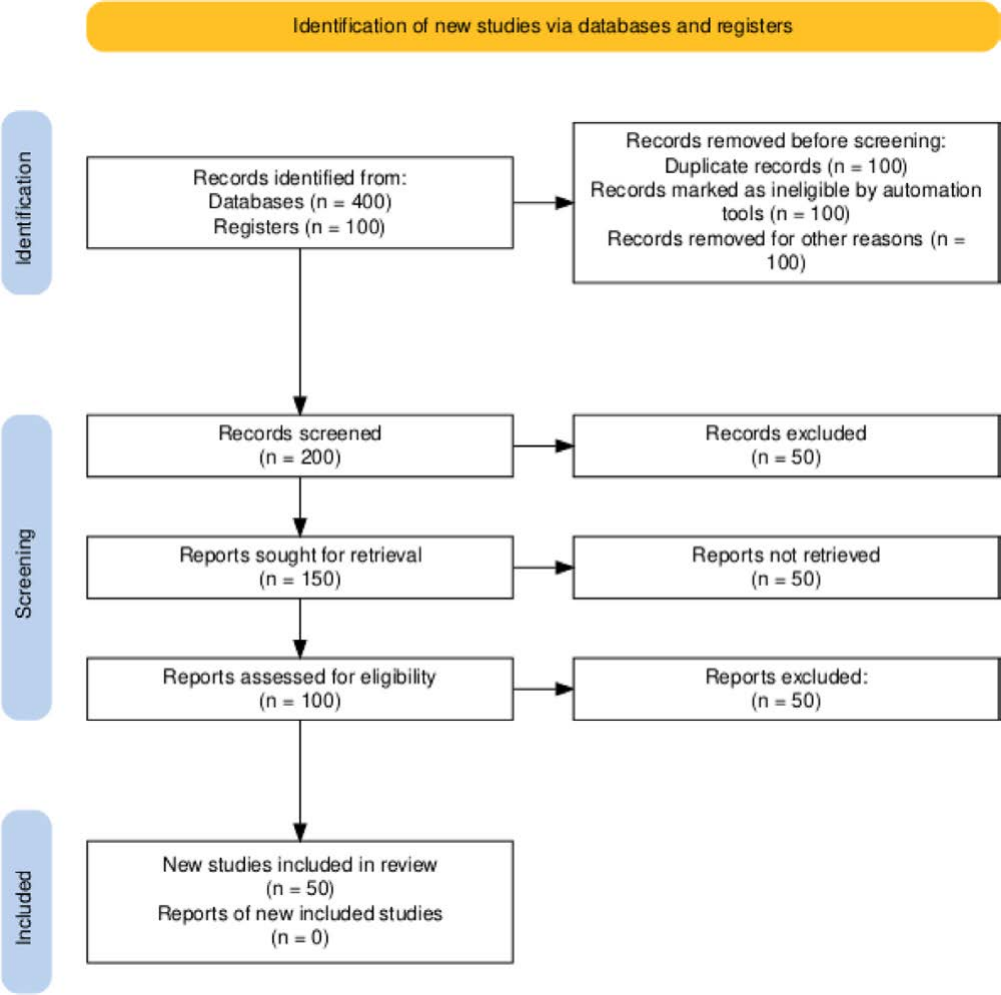
Flow diagram example and explanation. In our meta-analysis, we identified 500 records from databases (400) and registers (100). After removing 300 records due to duplicates, automation exclusions, and other reasons, we screened 200 records. Of these, 50 were excluded, and we sought full-text reports for the remaining 150 records, but 50 could not be retrieved. We assessed the eligibility of 100 full-text reports, excluding 50. Ultimately, 50 records met the inclusion criteria and were incorporated into the review.

The PRISMA flow diagram visually represents the study selection process, enhancing transparency and standardization in reporting systematic reviews and meta-analyses. Updates in PRISMA 2020 emphasize detailed reporting and the use of automation tools to improve methodological rigor (see Fig. 2).^[37]^

## 8. Data extraction

### 8.1. Data extraction overview

Data extraction should be performed by 2 independent reviewers to ensure accuracy and consistency. Any discrepancies between reviewers should be resolved through discussion, with a third reviewer involved if necessary. Use standardized forms and follow a clear protocol outlining the data to be extracted. Pilot test the forms with a small sample to refine the process, and train reviewers to maintain consistency. Document any deviations from the protocol to ensure transparency and reliability.

### 8.2. Training and pilot testing

Training data extractors is critical to ensure comprehension and consistent application of the extraction form. Conducting pilot testing of the extraction form with a subset of included studies helps refine its clarity and comprehensiveness. This process enhances the reliability of data extraction throughout the review process.^[31]^

## 9. Assessing study quality

Quality assessment tools in research were developed to systematically evaluate the methodological rigor and reporting quality of studies, ensuring the reliability and validity of their findings. These tools were created through a combination of expert consensus, literature review, and empirical testing. They typically include criteria that address various aspects of study design, conduct, analysis, and reporting.

For instance, the Cochrane Collaboration’s tool for assessing the ROB in randomized trials was developed by a panel of experts who identified key domains where bias could be introduced, such as selection, performance, detection, attrition, and reporting biases.^[48]^ These domains are then assessed using specific criteria to determine the overall ROB in a study. Similarly, tools like the Newcastle-Ottawa Scale (NOS) for nonrandomized studies were developed to evaluate the quality of observational studies.^[49]^ The NOS considers factors such as the selection of study groups, the comparability of groups, and the ascertainment of either the exposure or outcome of interest. Creating these tools often involves multiple stages, including identifying relevant quality indicators, pilot testing and refinement, and validation studies to ensure that the tools accurately measure what they are intended to.^[30]^ It is crucial to evaluate the data collected to ensure the validity and reliability of the results. This process includes developing a rigorous method for assessing the study’s quality and identifying potential bias risks. Study quality refers to the extent to which a study is free from methodological errors and can provide valid results.^[49]^ Evidence and results should be interpreted considering the quality of the included studies. The research quality encompasses the study’s conduct (its methodological quality) and its description (reporting quality and reproducibility). Poor methodological and reporting quality in primary studies included in the review may introduce bias and lead to spurious conclusions. Therefore, a valid assessment of study quality by 2 independent reviewers is essential to ensure accuracy and generalizability. An important aspect of methodological quality is the ROB in the included studies.^[50]^

The ROB in medical research refers to the chance that a study’s outcomes are affected by systematic factors that skew the conclusions away from the truth. These biases can occur at various stages of the research process, including design, data collection, analysis, or interpretation of the results. To conduct a comprehensive ROB assessment, it is essential to follow a structured approach: selecting tools specifically created for the study design you want to assess. These tools should demonstrate proven validity and reliability, focus on aspects of methodological quality (internal validity), and ideally be based on empirical evidence of bias.^[30]^ After obtaining the full-text articles, it is important to select the appropriate tools for evaluation. Compiling a detailed list of retrieved articles and organizing them by study type is crucial for efficient analysis. Table 4 displays commonly used tools for assessing ROB, aiding in the informed selection and systematic organization based on study design. For instance, Risk of Bias (ROB) tools like ROB2 assess biases in randomized controlled trials (RCTs), ensuring the reliability of study results.^[48]^ Risk of Reporting Bias tools (RRB), such as those under Risk of Bias In nonrandomized Studies (ROBINS), evaluate biases in nonrandomized studies of interventions (ROBINS-I) and exposures (ROBINS-E).^[51–53]^ A Measurement Tool to Assess Systematic Reviews (AMSTAR) evaluates the methodological quality of systematic reviews, while Grading of Recommendations Assessment, Development and Evaluations (GRADE) assesses the quality of evidence and strength of recommendations derived from systematic reviews. These tools are indispensable in promoting rigorous research practices and enhancing transparency in evidence synthesis across healthcare and scientific disciplines.^[54,55]^

**Table 4:** Commonly used tools for assessing ROB.

Tool	Full name/reference	Purpose/characteristics	Examples/usage
GRADE System^[55]^	Grading of Recommendations Assessment, Development, and Evaluation System	Provide a systematic approach for making clinical practice recommendations. The GRADE framework is not used to assess ROB within individual studies	Used by healthcare providers, guideline developers, and policymakers to make informed decisions about patient care based on the best available evidence.
RoB2^[48]^	Cochrane Risk of Bias Tool Version 2	Assesses ROB in randomized trials.	Applied in systematic reviews to evaluate the quality of included studies.
AHRQ^[56]^	Agency for Healthcare Research and Quality Methods Guide	Offers methodological guidance for evidence-based healthcare research.	Used in healthcare research to ensure rigorous methods in systematic reviews.
RRB^[53]^	RoBINS-I and RoBINS-E for umbrella reviews	Tools for assessing RoBINS-I and RoBINS-E.	Applied in umbrella reviews combining evidence from diverse study designs.
AMSTAR 2^[54]^	A MeaSurement Tool to Assess systematic Reviews 2	Evaluates the methodological quality of systematic reviews.	Used to appraise systematic reviews across various healthcare topics.
Newcastle-Ottawa^[49]^	Newcastle-Ottawa Scale	Rates quality of nonrandomized studies in meta-analyses.	Commonly used in meta-analyses of observational studies.
QUADAS ^[57]^	Quality Assessment of Diagnostic Accuracy Studies	Assesses the quality of diagnostic accuracy studies.	Applied in systematic reviews evaluating diagnostic test accuracy.
JADAD^[58]^	Jadad Scale	Rates quality of RCTs.	Commonly used in clinical trials to assess methodological quality.
STROBE^[59]^	Strengthening the Reporting of Observational Studies in Epidemiology	Provides guidelines for reporting observational studies.	Used by researchers to enhance transparency and quality of observational research reporting.
CONSORT^[60]^	Consolidated Standards of Reporting Trials	Guidelines for reporting randomized controlled trials.	Widely used by journals and researchers to improve transparency in reporting RCTs.
ROBINS-I^[53]^	Risk of Bias In nonrandomized Studies of Interventions	Tool to assess risk of bias in nonrandomized intervention studies.	Used in systematic reviews where randomized trials are unavailable or impractical.
ROBINS-E^[53]^	Risk of Bias In nonrandomized Studies – of Exposures	Tool to assess risk of bias in nonrandomized studies of exposures.	Applied in systematic reviews focusing on nonrandomized exposure studies.

Abbreviations: GRADE = grading of recommendations, assessment, development, and evaluations, ROB = risk of bias, RoBINS-I = risk of bias in non-randomized studies-interventions, RoBINS-E = risk of bias in non-randomized studies-exposures RCTs = randomized controlled trials.

Table 4 provides a concise overview of key tools used for assessing various types of studies and their primary characteristics.^[48,49,53–60]^ These tools play crucial roles in evaluating research quality and bias across different methodologies. The next step in assessing bias involves understanding distinct categories such as selection, performance, attrition, detection, and selective outcome reporting biases. For a detailed discussion on these types of bias and their assessment, refer to the Cochrane Handbook for Systematic Reviews of Interventions, which provides comprehensive guidance on identifying and addressing these biases.^[61]^ This approach ensures a thorough evaluation, allowing separate ratings for each outcome to account for variations in detection and reporting biases specific to each outcome measure. It’s crucial to evaluate bias based on study design and execution rather than solely on reporting quality. Poorly reported studies may lead to an unclear ROB assessment. Avoid aggregating bias assessments into a single composite score. Instead, it’s more practical to classify bias as “low,” “medium,” or “high.”^[30]^ Establishing predefined methods for overall categorization of study limitations is essential and should be clearly documented beforehand. This systematic approach helps ensure the internal validity of studies included in the systematic review, thereby enhancing the reliability of conclusions drawn from the review based on robust, high-quality evidence.^[30]^

## 10. Data synthesis and statistical analysis

### 10.1. Data synthesis and statistical analysis overview

Once we have made our data extraction and our quality assessment, we can start with the data synthesis, which can be qualitative synthesis or narrative summary, and quantitative synthesis, specifically meta-analysis, and these represent contrasting approaches for summarizing research findings. Narrative synthesis involves a descriptive aggregation of study results, focusing on themes, patterns, and commonalities without statistical pooling.^[61]^ It emphasizes understanding the context and methodologies of included studies, often incorporating subjective interpretation and expert judgment. In contrast, meta-analysis quantitatively combines data from multiple studies to calculate an overall effect size and its confidence interval.^[61]^ In systematic reviews, the choice between qualitative synthesis and quantitative synthesis, particularly through meta-analysis, hinges on the nature of available data and research objectives. Qualitative synthesis involves systematically reviewing and narratively summarizing findings across studies without statistical aggregation, focusing on themes, patterns, or concepts. This approach is suitable when studies exhibit heterogeneity or when quantitative data cannot be feasibly combined due to methodological or substantive differences. On the other hand, quantitative synthesis and meta-analysis statistically pool data from multiple studies to generate summary effect estimates, offering increased precision when studies are sufficiently similar. This method is advantageous for providing quantitative summaries of evidence and estimating overall effects or associations. The decision between qualitative and quantitative synthesis methods should align with the review’s goals and the homogeneity of available data.^[61]^

Meta-analysis utilizes various statistical software tools (to compute effect sizes and their associated 95% confidence intervals (CI), often represented graphically through forest plots (see Table S3, Supplemental Digital Content, https://links.lww.com/MD/O720, which provides examples and details about the characteristics of different tools for data synthesis).^[62–68]^ These plots provide a visual summary of study outcomes, and the endpoints indicating the CI bounds.

R is a programming language and software environment designed for statistical computing and graphics. It was created by Ross Ihaka and Robert Gentleman at the University of Auckland, New Zealand, in the early 1990s. R was developed to provide an open-source alternative to proprietary statistical softwares. Its development has been community-driven, with contributions from statisticians, researchers, and developers worldwide. R’s popularity has grown significantly due to its flexibility, vast package ecosystem, and active community support, making it a powerful tool for statistical analysis in academia, industry, and research.^[69]^ Review Manager (RevMan), endorsed by the Cochrane Collaboration, is widely used for systematic reviews and meta-analyses. It facilitates the calculation of effect sizes and generates forest plots to visualize the results. These plots help to interpret the size and accuracy of effects across studies.^[63]^

At this point, systematic reviews and meta-analyses follow the same steps. However, not all systematic reviews require a meta-analysis, although a meta-analysis is always part of a systematic review. To perform a meta-analysis, it is important to carefully consider the statistical model fixed-effects (FE) or random-effects (RE).

FE models assume that all studies estimate a single common effect, which is appropriate when the studies are highly similar in terms of participants, interventions, and outcomes. In contrast, RE models allow for the possibility that true effects vary across studies, accounting for both within-study error and between-study heterogeneity. These distinctions make RE models more suitable for data with notable variability among studies. Choosing between these models requires a nuanced approach. While heterogeneity metrics, such as *I*^2^ or Cochran *Q*, are commonly referenced to guide model selection, they should not serve as the sole basis for decision-making. For instance, high heterogeneity does not necessarily invalidate the use of FE models if the primary goal is to estimate an effect in a specific context. Conversely, RE models distribute weights more evenly across studies and may generalize findings to broader contexts, but they can overemphasize smaller studies with high variance.^[70]^ Key considerations in model selection include whether the studies are sufficiently similar to justify a common effect FE or better represented by a distribution of effects RE; the consistency of designs, participant populations, and interventions across studies; whether the aim is to generalize findings beyond the included studies favoring RE or focus narrowly on a specific setting or population favoring FE; and assessing how model assumptions affect results using sensitivity analyses. Comparing FE and RE results can provide clarity on the firmness of conclusions It is recommended to check the cap 10.10.4 of the Cochrane Handbook for a comprehensive discussion on these considerations, including the potential for misinterpretation if the model assumptions are not met.^[61]^ Proper sensitivity analyses and a clear articulation of the rationale for model selection further enhance the validity and interpretability of the results. For additional information about when to use different types of summary measures in meta-analysis (e.g., MD, SMD, OR, RR), we recommend referring to the Cochrane Handbook for Systematic Reviews of Interventions.^[61]^

### 10.2. Heterogeneity

Heterogeneity between studies refers to differences in effects that may be due to chance or other factors. If all studies were conducted in exactly the same way, then chance would be the only explanation for differences in effect estimates.^[2]^

In reality, studies are conducted in slightly different ways, so differences in effects are due to chance and other factors. This heterogeneity is evident by visual inspection of the pooled data, and nonoverlapping CI suggest (but do not confirm) the presence (but not the extent) of heterogeneity.^[2]^

The *I*^2^ statistic, as proposed by Higgins and Thompson, is derived from the *Q* statistic of the Cochran test and the number of studies. It has a range from negative values to 100%, but when negative, it is considered to be 0. The *P*-value of *I*^2^ is equivalent to the *P*-value of *Q*^2^. According to Higgins et al, an *I*^2^ value closer to 0% suggests nonheterogeneity among studies, while values closer to 25%, 50%, and 75% indicate low, moderate, and high heterogeneity among studies, respectively.^[71]^

The Cochran *Q*-test evaluates whether the studies in a meta-analysis are homogeneous, assuming that they have no differences. A higher *Q*-value indicates increased heterogeneity. However, one limitation is that *Q*-value can range from 0 to infinity, making interpretation challenging. Another drawback is that the test may lack power when there are a small number of studies, it may produce false identifications of heterogeneity. Additionally, the test provides a *P*-value, which indicates the significance of the heterogeneity compared to zero.^[72]^ It is recommended that researchers attempt to investigate potential sources of heterogeneity using methods such as subgroup analysis, meta-regression, sensitivity analysis, among others, to better understand the observed variabilities in the results and ensure the validity of the conclusion.

### 10.3. Sensitivity analyses

Sensitivity analysis aims to study the influence of each study on the weighted effect estimate, and thus the robustness or stability of the final measure obtained. This analysis involves repeating the meta-analysis as many times as there are studies, each time omitting one study and using all the remaining ones. If the results of the various meta-analyses are similar, meaning the effect has the same direction, magnitude, and statistical significance, the results can be concluded as robust. Otherwise, the estimator would not be robust, requiring caution in interpreting the results or possibly generating new hypotheses.

A noteworthy point is that when high-quality and precise studies are removed, the magnitude of the final result expands, helping to identify such studies. This can be graphically represented through a forest plot, known as an influence plot, which graphs the weighted effect for each analysis, always excluding one study. Sensitivity analysis can also be used to study the influence on meta-analysis results of certain aspects related to the validity of studies, such as excluding studies that do not meet a certain quality threshold, unpublished works, or studies with uncertainty about meeting selection criteria. Additionally, it is possible to calculate the number of unpublished zero-effect studies needed to change the direction of a positive conclusion obtained from a meta-analysis. This is known as Fail-Safe N, using methods by Rosenthal (1979) and Orwin (1983). If this number is very high, it is considered that the probability of publication bias significantly altering the results is low, and the differences suggested by the meta-analysis are accepted.^[73–75]^

### 10.4. Publication bias

When primary studies are not accessible, a review might encounter obstacles regardless of its best efforts to include every relevant study. Studies with positive outcomes tend to be published more often than those with negative or null findings, although various factors influence publication decisions.^[2]^ Publication bias results from this phenomenon. There are several ways to assess the possibility of publication bias. The use of funnel plots is one popular strategy that will be explained more in-depth in further sections. These graphs show the scatter of all the studies that were part of the meta-analysis, with the standard error plotted on the y-axis and the effect size plotted on the x-axis.^[2]^ Missing research in the literature is indicated by an asymmetrical funnel diagram.^[6]^ But only when the meta-analysis includes more than 10 trials will this approach be successful.^[2]^

However, it can be difficult to clearly identify asymmetry and even it could be visually present this method is subjective to a graphical representation. As a result, formal statistical methods have been developed, including Egger regression.^[6]^ Egger regression analyzes whether unpublished trials with lower effects continue to be unpublished, and whether smaller studies show bigger effect sizes than would be predicted by chance.^[8]^ This test evaluates the association between effect sizes and their sample variances; a substantial correlation indicates publication bias. If there is no publication bias, the regression intercept should ideally equal zero.^[14]^ Nevertheless, it’s essential to remember that variables other than publication bias, including study heterogeneity, selective outcome reporting, or chance, can also result in funnel plot asymmetry.^[6]^

Sensitivity tests can be carried out after determining a funnel plot’s asymmetry. The trim-and-fill approach is one way to achieve this.^[10]^ This 2-step approach intends to detect publication bias and correct results accordingly. Starts by 1) Trimming which produces a symmetrical plot by excluding small studies, and then estimates an adjusted summary effect by considering just the larger studies. And then 2) Filling to recreate the funnel plot around the corrected summary estimate by substituting the studies that were removed with mirror-image “filled” studies—that is, studies that are assumed to be unpublished—are then added.^[6,14]^ Although thorough reporting and discussion are necessary to combat publication bias, the study’s publication shouldn’t be hindered by it.^[15]^

## 11. Presenting results

### 11.1. Presenting results overview

Once we have performed our meta-analysis and understood the results, the next step is to present them. The results section of a meta-analysis includes several key components. One of these is the summary of estimate, also known as pooled estimate, which represents the average outcome derived from the different studies.^[6]^ It can be calculated using parameters such as mean differences (MDs), weighted mean difference (WMDs), standardized mean differences (SMDs), proportions, differences in proportions, relative risks (RRs), and odds ratios (ORs), among others (see Table 5).^[76–80]^ The summary of estimate is typically reported along a 95% confidence interval (CI) range, which means that there is a 95% probability that the parameter falls within this range. For this reason, confidence interval can be described as the “range of possible values for the actual magnitude of the effect”.^[76]^

**Table 5:** Parameters of summary estimate.

Parameter	Meaning
Mean differences^[76]^	Measures the difference between the mean value in 2 groups, experimental group and control group. Assesses the effectiveness of the experimental group. The change of means and final means can be combined.
Standardized mean differences^[76,77]^	Used when all studies evaluate the same outcome but measure it with different instruments or ways. SMDs equals to the mean difference of the outcomes divided by its standard deviation. The most common ones are Cohen *d*, Hedges’ *g*, and Glass’ delta.
Proportions^[78]^	Number of cases divided by the total population number.
Differences in proportions^[78]^	Difference between the proportion of a specific outcome between 2 groups.
Relative risk^[79]^	Risk of event in experimental group divided by risk of event in control group. RR > 1.00 indicates increased risk.
Odds ratios^[79]^	Odds of event in experimental group divided by odds of event in control group. OR > 1.00 indicates increased risk.
Hazard ratio^[80]^	Risk of an event or disease relative to an exposure. Ratio of the chance of an event occurring in treatment group over the chance of the event in a control group.

Abbreviations: OR = odds ratios, RR = relative risk, SMDs = standardized mean differences.

Furthermore, the investigator must review the heterogeneity value. When heterogeneity is present, techniques such as subgroups and meta-regression can be used to address it (see Table 4, Supplemental Digital Content, https://links.lww.com/MD/O720, which describes statistical tools to describe heterogeneity)^[61]^

Additionally, single-arm trials are a common clinical trial design in which all participants have a specific medical condition and receive the experimental therapy, followed by a period of observation. These trials are used to collect data on the efficacy and safety of the treatment.^[81]^

Diagnostic test accuracy studies are a method used to enhance the study’s validity level. They measure accuracy parameters such as sensitivity, specificity, positive and negative likelihood ratios, diagnostic odds ratio (DOR). The summary receiver operating characteristic (SROC) curve shows the relation of the sensitivities and specificities of the studies. There are 2 types of SROC curve models: the Moses-Littenberg SROC Curve and the Hierarchical models, including the bivariate model and Hierarchical Summary Receiver Operatic Characterictic model (HSROC). The Moses-Littenberg SROC curve does not account for study heterogeneity; whereas the HSROC model, a statistical approach that generate a summary of the SROC curve, is preferred because it considers within-study variabilities and estimates heterogeneity, making it more effective.^[82]^ For this reason, some of the most commonly used software programs include open-access program language like R, as well as commercial statistical software such as SAS or STATA).^[62,64,83]^

### 11.2. Visual representation

Furthermore, tables and graphs are essential tools for the analysis and interpretation of the results, since they organize the information in an understandable and concise way. It allows the presentation of large numbers of individuals in a well-organized and appealing method, allowing the reader to comprehend it efficiently.^[84]^ Therefore, the visualization of tables and graphs are useful for summarizing and comparing data between studies, making forest and funnel plots the gold standard figures in meta-analysis.^[85]^

### 11.3. Interpreting forest plots results

**Figure 3.**
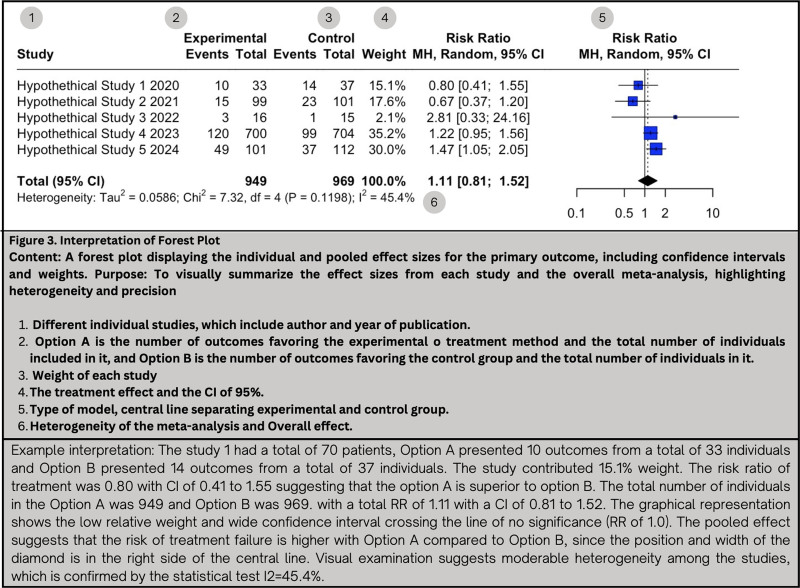
Interpretation of forest plot. A forest plot displaying the individual and pooled effect sizes for the primary outcome, including CI and weights. Purpose: To visually summarize the effect sizes from each study and the overall meta-analysis, highlighting heterogeneity and precision. 1. Different individual studies, which include author and year of publication. 2. Option A is the number of outcomes favoring the experimental o treatment method and the total number of individuals included in it, and Option B is the number of outcomes favoring the control group and the total number of individuals in it. 3. Weight of each study. 4. The treatment effect and the CI of 95%. 5. Type of model, central line separating experimental and control group. 6. Heterogeneity of the meta-analysis and Overall effect. Example interpretation: The Study 1 had a total of 70 patients, Option A presented 10 outcomes from a total of 33 individuals and Option B presented 14 outcomes from a total of 37 individuals. The study contributed 15.1% weight. The risk ratio of treatment was 0.80 with CI of 0.41 to 1.55 suggesting that the option A is superior to option B. The total number of individuals in the Option A was 949 and Option B was 969. with a total RR of 1.11 with a CI of 0.81 to 1.52. The graphical representation shows the low relative weight and wide confidence interval crossing the line of no significance (RR of 1.0). The pooled effect suggests that the risk of treatment failure is higher with Option A compared to Option B, since the position and width of the diamond is in the right side of the central line. Visual examination suggests moderable heterogeneity among the studies, which is confirmed by the statistical test *I*^2^ = 45.4%. CI = confidence interval, RR = relative risk.

In meta-analysis, forest plots are visual representations that provide information about the precision, weight, effect estimates, and CI of each study (see Fig. 3).^[61,85]^ They consist of 2 axes: X and Y. The Y-axis (vertical line), also known as central trend axis, represents the point at which there is no difference between interventions, with the RR = 1 or the Mean Difference = 0. On the other hand, the X-axis (horizontal line) shows the estimated effect size for an outcome.^[77]^ The Y-axis divides the X-axis in half, with the left side indicating support for one intervention (experimental or treatment) and the right side supporting another intervention (control). In forest plots, each individual study is represented by a geometrical figure (usually a square or circle), a horizontal line, and a vertical line in the middle of the square. The size of the geometrical figure indicates the estimated effect of the individual study; the larger the square, the greater the weight of the study. The vertical line represents the individual result of each study, and its position on the X-axis reflects the direction of the intervention in that study. The horizontal line represents the confidence interval for each study.^[70]^ The precision of each study is shown by a 95% confidence interval.^[85]^ If the entire line is on one side of the Y-axis, it indicates a statistically significant trend in favor of that specific intervention (*P* < .05). However, if the line crosses or touches the Y-axis, it suggests no statistically significant difference between the 2 interventions (*P* > .05).^[70]^

Another feature in forest plots is the diamond or rhombus, typically located beneath all the studies, which represents the combined effect of all the studies. The center of the diamond indicates the result of the meta-analysis and shows which intervention the results favor based on its position. The width of the diamond reflects the confidence interval of the meta-analysis, and if it crosses or touches the Y-axis, it suggests that there is no statistically significant difference between both interventions (*P* > .05).^[70]^

### 11.4. Interpretation example for forest plot

Suppose our meta-analysis is assessing the effect of a new drug on reducing blood pressure (see Fig. 3). Each study shows its weight and its confidence interval. In this case, all the studies are not statistically significant because the confidence line (horizontal line) touches the Y-axis, except for study 5. Additionally, the overall diamond favors control group but it is not statistically significant because the figure touches the Y-axis. As well, the figure shows that *I*^2^ = 45%, which means that studies have a moderate heterogeneity between them.

### 11.5. Interpreting funnel plots results

**Figure 4.**
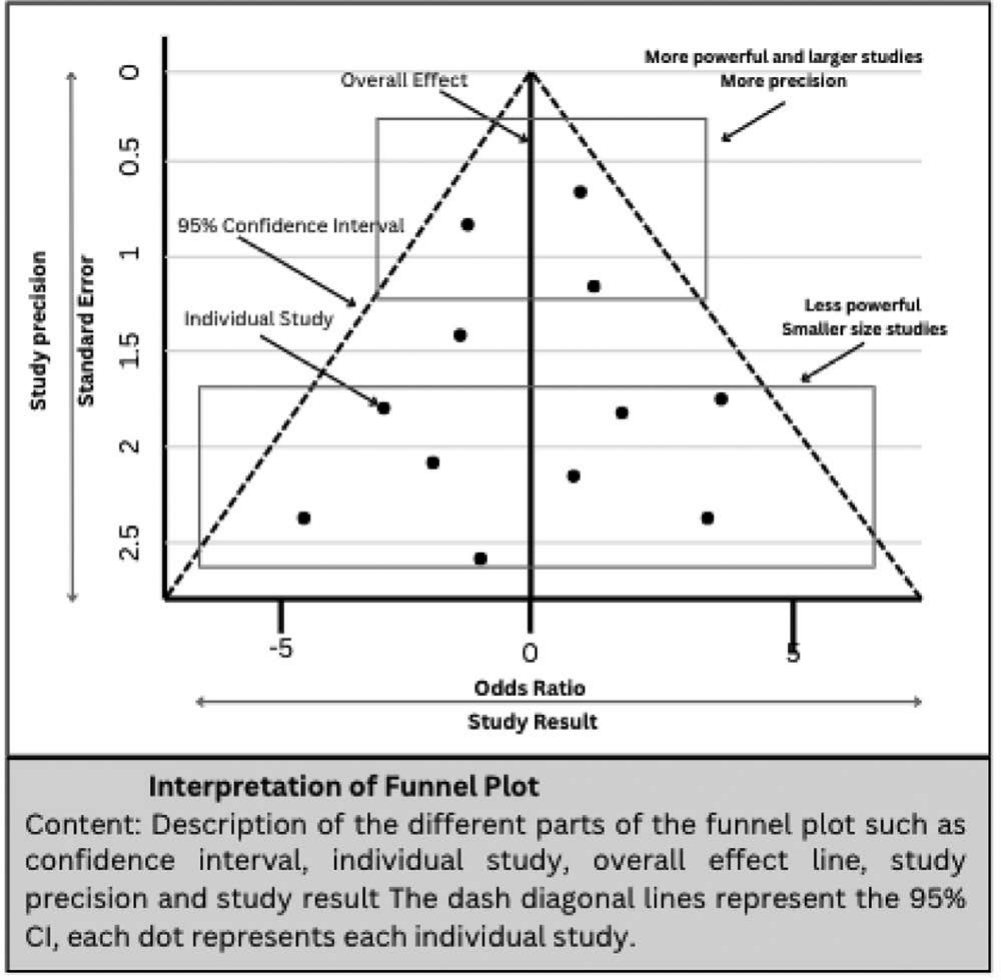
Interpretation of funnel plot. Description of the different parts of the funnel plot such as confidence interval, individual study, overall effect line, study precision and study result The dash diagonal lines represent the 95% CI, each dot represents each individual study.

Additionally, funnel plots are used to measure publication bias and variance among studies. It is a scatter plot that compares the effect size (X-axis or horizontal axis) like Standardized Mean Difference, ORs or Risk Ratios with a measure of their precision (Y-axis or vertical axis) like sampling variance or standard error.^[75]^ The diagonal lines represent the 95% Confidence Interval (see Fig. 4). Each dot in the graph symbolizes an individual study and should be expected to lie under the diagonal lines representing absence of heterogeneity. Smaller studies are scattered widely at the bottom of the plot, and larger studies tend to have higher precision and are gathered near the top of the plot.^[86]^

**Figure 5.**
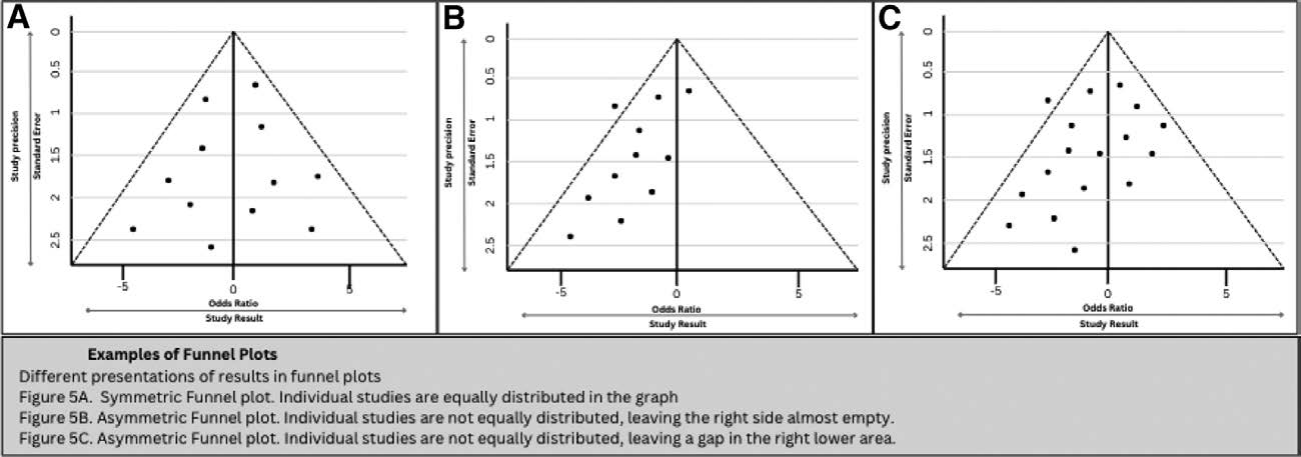
Examples of funnel plots. Different presentations of results in funnel plots. (A) Symmetric Funnel plot. Individual studies are equally distributed in the graph. (B) Asymmetric Funnel plot. Individual studies are not equally distributed, leaving the right side almost empty. (C) Asymmetric Funnel plot. Individual studies are not equally distributed, leaving a gap in the right lower area. CI = confidence interval.

A symmetrical funnel plot should look like a pyramid and it is an equal distribution of the studies around the central line (see Fig. 5A). On the other hand, if the funnel plot is asymmetric, it means that publication bias can exist.^[75]^ Asymmetry can be exhibited in the funnel plot when there is an unequal distribution of studies (see Fig. 5B) or if there is a gap in one of the regions (see Fig. 5C).^[86]^ If small studies have larger effects, asymmetry can also occur.

Numerous statistical tests for publication bias in funnel plot were proposed, such as Egger regression test, Begg rank test, and Trim and Fill method. Egger test involves doing a linear regression of the standardized effect size on the precision of the study. The Begg rank test evaluates the association between the effect size and the sampling variance.^[87]^ The Trim and Fill method consists on removing smaller studies to have a symmetric funnel plot and then predict the adjusted summary effect considering the larger studies.^[88]^

### 11.6. Interpreting results importance

Meta-analysis is on the top of hierarchy pyramid of evidence. Since it includes the results of several individual studies, it plays a major role in evidence-based medicine. Clinically, it is relevant because it shows the estimate of treatments or risk factors of different diseases. Meta-analysis impacts the patient’s outcome due to its capacity to compare interventions. In the area of policy-making, they bring support and recommendations in the making or modification of clinical guidelines and protocols, diagnostic criteria and treatment methods.^[85]^ Nevertheless, the last step is the GRADE system, a systematic approach for grading the quality of evidence at an outcome level and for producing clinical practice recommendations. For comprehensive guidance on applying the GRADE framework, consult the official GRADE Handbook, which outlines the principles and processes for evaluating evidence quality and recommendation strength.^[55]^

## 12. Common errors

Several common errors can occur when conducting a meta-analysis. One of the most frequent mistakes involves data entry or transposition errors, which can significantly impact results.^[89]^ Researchers should be vigilant during this step, and it is recommended to have 2 independent reviewers perform the data entry, followed by a double-check of the database to prevent errors in the analytic step of the data. The author responsible for carrying out the meta-analysis should be cautious of the database and ensure that the reported values are entered in the same units, for example entering the whole database using SD with CI, watching out of not having a median value. Additionally, all effect size data must be reported consistently to prevent discrepancies caused by mixing different statistical measures. Also, standard error means (SEMs) are sometimes mistakenly used interchangeably with SD values, this leads to an overestimation of the effect size and narrow effect sizes CI.^[90]^ Therefore researchers should be cautious when undergoing the data extraction to prevent confusing standard deviation (SD) values with SEM values. Other examples include not including a minus sign when entering the values.^[89]^ Double counting is another critical error, occurring when the same study participants are inadvertently included multiple times in an analysis. This can happen when a systematic review incorporates data from previous reviews without properly distinguishing between unique and duplicated studies or when multiple study arms are included as separate data points. Handling outliers correctly is also essential. While there is no universal definition of an outlier, one general approach defines them as values more than 3 SDs from the mean or more than 1.5 times the interquartile range from the median.^[90]^ After identifying an outlier, authors must confirm that the information is accurate, and in this case, correct the database. If the information is correct, then analyze the data with or without the outlier to see the influence of the study within the whole meta-analysis. Other mistakes include, not weighing the studies by the inverse of the within-study variance, which could result in all studies within a meta-analysis being considered equally regardless of the size of the study, thus resulting in biased conclusions.^[90]^ Researchers should be careful not to overlook or underestimate publication bias, as it can lead to an overestimation of effect sizes and distort conclusions.^[91]^ Study selection is important within a meta-analysis, prioritizing RCT trials, but also utilizing other control trials, cohort, and case-control studies, which are usually the type of studies that are used in a meta-analysis. Erroneous study selection, such as including another type of study, can draw incorrect conclusions when undergoing mathematical analysis. It is extremely important to prioritize studies with control groups, to provide a between-group comparison of the intervention with its control. Yet, another common mistake is the inappropriate pooling of control treatments, this happens when authors try to compare a new treatment modality with several treatment controls. For example, the novel use of ketamine in sickle cell disease vs standard treatment modality, which includes multiple treatments such as nonsteroidal anti-inflammatory drugs (NSAIDS), opioids, and acetaminophen, and there’s not a well-defined standard treatment modality, therefore the results will determine the difference between ketamine and multiple different treatments, making the conclusion more nuanced. This type of error is also related to the methodology, in which the definition of standard treatment should be well defined. The methodology should always be well defined and with a structured PICO question before starting the project, this ensures that the search strategy is also well done, as there are examples of studies that use terms interchangeably when they are not. Furthermore, the PRISMA flow chart must follow the selection process logically and adequately. Additionally, interpretation of results given in a meta-analysis should be done in a self-critical manner and with transparency regarding the limitations of the study.

Errors in meta-analysis are alarmingly common, it was found that in 20 of the most cited and influential meta-analyses of the last 20 years, 75% included at least one error.^[90]^ Due to the importance of meta-analysis in scientific influence and clinical decisions it is necessary to be aware of these mistakes and to prevent producing these common mistakes when conducting a meta-analysis.

## 13. Conclusion

In conclusion, systematic reviews and meta-analyses are indispensable tools in contemporary medical research and evidence-based practice, providing robust, empirically supported answers to critical clinical questions. These methodologies enhance the reliability of findings by systematically integrating data from multiple studies, thereby reducing bias and increasing the accuracy of estimates. Formulating a well-defined research question using frameworks like PICOTS, employing comprehensive literature search strategies, and rigorously assessing study quality are essential steps to ensure the validity and reproducibility of systematic reviews and meta-analyses. Proper data extraction, protocol adherence, and the application of suitable statistical methods for data synthesis are critical to deriving meaningful conclusions. Visual tools such as forest and funnel plots aid in the interpretation and presentation of results, offering clear insights into the effects and potential biases in the studies analyzed. Despite challenges such as publication bias and heterogeneity, systematic reviews and meta-analyses remain at the pinnacle of the evidence hierarchy, profoundly influencing clinical guidelines, policy-making, and patient care. Understanding and addressing common errors in these processes further ensure the integrity and impact of the research. By adhering to rigorous methodological standards, researchers can produce high-quality, reliable evidence that advances medical knowledge and improves healthcare outcomes.

## Author contributions

**Conceptualization:** Ernesto Calderon Martinez.

**Investigation:** Patricia E. Ghattas Hasbun, Vanessa P. Salolin Vargas, Oxiris Y. García-González, Mariela D. Fermin Madera, Diego E. Rueda Capistrán, Thomas Campos Carmona.

**Methodology:** Ernesto Calderon Martinez.

**Project administration:** Ernesto Calderon Martinez.

**Resources:** Ernesto Calderon Martinez, Patricia E. Ghattas Hasbun, Vanessa P. Salolin Vargas, Oxiris Y. García-González, Mariela D. Fermin Madera, Diego E. Rueda Capistrán, Thomas Campos Carmona.

**Supervision:** Ernesto Calderon Martinez, Patricia E. Ghattas Hasbun.

**Validation:** Ernesto Calderon Martinez.

**Visualization:** Patricia E. Ghattas Hasbun.

**Writing – original draft:** Patricia E. Ghattas Hasbun, Vanessa P. Salolin Vargas, Oxiris Y. García-González, Mariela D. Fermin Madera, Diego E. Rueda Capistrán, Thomas Campos Carmona.

**Writing – review & editing:** Ernesto Calderon Martinez, Patricia E. Ghattas Hasbun, Vanessa P. Salolin Vargas, Thomas Campos Carmona, Camila Sanchez Cruz, Camila Teran Hooper.

## Supplementary Material


