# Supplementary material for: A comprehensive guide to conduct a systematic review and meta-analysis in medical research
Source: Medicine (Baltimore). 2025 Aug 15;104(33):e41868. doi: 10.1097/MD.0000000000041868 (PMC12366998; doi:10.1097/MD.0000000000041868)
Supplement: Supplementary file 1 [file medi-104-e41868-s001.pdf]

Supplemental Digital Content, Table 1, which compares PRISMA-p and Cochrane. [30,37]

**Section/Topic**	**PRISMA-P Checklist Item**	**Cochrane Protocol Section**
**Administrative Information**		
**Title**	1a Identify as protocol of a systematic review	Title
**Update**	1b Specify if updating a previous review	-
**Registration**	2 Name of registry and registration number	Registration and protocol
**Authors**	3a Authors' details (name, affiliation, contact info)	Authors
**Contributions**	3b Describe authors' contributions, identify guarantor	Contributions of authors
**Amendments**	4 Plan for documenting protocol amendments	-
**Support**		
**Sources**	5a Sources of financial or other support	Sources of support
**Sponsor**	5b Funder/sponsor name	-
**Role of sponsor/funder**	5c Describe roles in protocol development	-
**Introduction**		
**Rationale**	6 Describe rationale for the review	Background

**Objectives**	7 Explicit statement of review question (PICO)	Objectives
**Methods**		
**Eligibility criteria**	8 Study and report characteristics (PICO, study design)	Criteria for selecting studies
**Information sources**	9 Planned information sources (databases, grey literature)	Search methods for identification
**Search strategy**	10 Detailed search strategy for at least one database	-
**Study records**		
**Data management**	11a Mechanism for managing records and data	Data, code and other materials
**Selection process**	11b Process for selecting studies	Criteria for selecting studies
**Data collection process**	11c Method for extracting data from reports	Data collection and analysis
**Data items**	12 Variables and data assumptions	Outcome measures
**Outcomes and prioritization**	13 List and prioritize outcomes	Outcome measures
**Risk of bias**	14 Methods for assessing risk of bias	-
**Data synthesis**	15a Criteria and methods for data synthesis	Data collection and analysis
**Additional analyses**	15b Planned additional analyses	-
**Type of summary**	15d Type of summary planned	-
**Meta-bias(es)**	16 Assessment of meta-bias	-

**Evidence strength**	17 Assessment of evidence strength (GRADE)	-
**Consumer Involvement**		
**Consumer involvement**	-	Consumer involvement
**Other Supplementary Materials**		
**Declarations of interest**	-	Declarations of interest
**References**	-	References
**Figures and Tables**	-	Figures and Tables

Supplemental Digital Content, Table 2, which compares different platforms for registering systematic review protocols.

Platform	Description	Advantages
**PROSPERO** [39]	First prospective register for systematic reviews, focuses on health-related reviews.	Comprehensive platform for protocol registration.
**Research Registry** [40]	Dedicated to health and medicine, facilitates registration of systematic reviews.	Promotes transparency and prevents research duplication.
**Open Science Framework** [41]	Versatile repository across disciplines, promotes open access and collaboration.	Facilitates open access and collaboration between disciplines.
**Figshare and VTechWorks** [42,43]	General-purpose repositories accommodating systematic review protocols.	Enhances accessibility and visibility of research outputs.

Supplemental Digital Content, Table 3, which describes different tools for data synthesis.

Software	Characteristics	Pros	Cons
**R** [62]	Open-source programming language and environment for statistical computing and graphics.	Free, extensive libraries (packages) for various statistical analyses. Flexible and customizable.	Steeper learning curve for beginners. Requires coding skills for advanced use.
**Rev Man**[63]	Software for conducting Cochrane systematic reviews and meta-analyses.	Specifically designed for systematic reviews. Standardized analysis methods.	Limited flexibility outside systematic reviews. Less suitable for complex analyses.
**Stata**[64]	Statistical software for data analysis, visualization, and modeling.	User-friendly interface. Comprehensive statistical capabilities.	Costly for individual licenses. Limited support for non-linear modeling.
**JASP** [65]	Free and open-source statistical software with a focus on Bayesian statistics.	User-friendly interface. Offers both Bayesian and frequentist analyses.	Less extensive package ecosystem compared to R.
**Jamovi** [66]	Free and open-source statistical software with a GUI similar to SPSS.	Easy-to-use GUI. Designed for users transitioning from SPSS.	Limited advanced statistical modeling compared to R or Stata.
**SPSS** [67]	Statistical software for data analysis, modeling, and reporting.	User-friendly GUI. Extensive statistical functions.	Expensive licenses. Less flexible for custom analyses compared to R.
**Comprehensive Meta-analysis Software** [68]	Standalone program for diverse meta-analyses with user-friendly tools and	User-friendly, versatile for various study designs, efficient with step-by-step guidance, and well-supported with tutorials.	Relatively expensive, less customizable for advanced users, and limited to desktop use.

	comprehensive outputs.		

Supplemental Digital Content, Table 4, which describes statistical tools to test heterogeneity. [61]

Statistical tool	Characteristic
**Subgroups**	Dividing all the participant data into groups to make comparison between them. Used when variable is categorical.
**Meta-regression**	Extension of subgroup analyses where the outcome variable (or effect estimate) is obtained by the explanatory variables or also known as covariates (characteristics that can influence the size of intervention effect).
